# Supplementary material for: SensibleSleep: A Bayesian Model for Learning Sleep Patterns from Smartphone Events
Source: PLoS One. 2017 Jan 11;12(1):e0169901. doi: 10.1371/journal.pone.0169901 (PMC5226832; doi:10.1371/journal.pone.0169901)
Supplement: S1 Appendix — (PDF) [file pone.0169901.s002.pdf]

S1 Poisson vs Power Law Distribution for screen events. Understanding the mechanisms behind the rhythms of human activity is a very active area of research. Many studies have reported that human dynamics follow power-law distributions, and different mechanisms have been suggested to be behind the emergence of heavy tailed behavior, such as circadian rhythm [1], mixtures of multiple agents [2, 3], cascading Poisson processes [4], priority queues [5] and burstiness [6]. Even if the underlying events in our case are not all of a communicative nature (i.e. checking the clock or starting an app), it is reasonable to check whether the actual distribution of the inter-events times are better described by a Power Law function such as the Pareto distribution:

$$f(x;\alpha,x_m) = \begin{cases} \frac{\alpha x_{\mathrm{m}}^{\alpha}}{x^{\alpha+1}} & x \geq x_{\mathrm{m}}, \\ 0 & x < x_{\mathrm{m}}. \end{cases}$$

or by an Exponential distribution, which would be expected if the underlying process is a Poisson process:

$$f(x;\lambda) = \begin{cases} \lambda e^{-\lambda x} & x \geq 0 \\ 0 & x < 0 \end{cases}$$

A power law distribution holds a linear relation between $log(x)$ and $log(y)$ whereas an exponential distribution holds a linear relation between $log(y)$ and x. Distributions of the inter-event times belonging to the users of Dataset B do not reveal a linear relation (a straight line) in neither a semilog nor a log-log graphical representation: compared to an exponential distribution the tail is longer, and compared to a power law, the tail is less broad.

Instead, in order to compare which of the two models has the better fit to the actual distributions, the pairwise difference between the Akaike Information Criterion (AIC – similar to the log likelihood ratio adjusted for the degrees of freedom), can be calculated between a maximum likelihood estimated (MLE) exponential vs Pareto distribution, for each user. The resulting distribution of the pairwise AIC differences is seen in Fig 1.

In total, 92.6% (in blue) of the the N=324 users show a lower AIC score for the MLE exponential distribution vs the Pareto distribution. Hence, there appear to be no strong arguments in favor of a power law distribution vs an exponential in the dataset as such, and it is reasonable to use the weakly informative assumption of a Poisson process.

As the event times here include the sleep periods which by nature hold longer event times and thus contribute to an extended tail, it can be expected that the distribution of the wake events alone may be even more in favor of the exponential distribution compared to the power law distribution.

References

1. Malmgren RD, Stouffer DB, Motter AE, Amaral LA. A Poissonian explanation for heavy tails in e-mail communication. Proceedings of the National Academy of Sciences. 2008;105(47):18153–18158.

2. Malmgren RD, Hofman JM, Amaral LA, Watts DJ. Characterizing individual communication patterns. In: Proceedings of the 15th ACM SIGKDD international conference on Knowledge discovery and data mining. ACM; 2009. p. 607–616.

3. Hidalgo R CA. Conditions for the emergence of scaling in the inter-event time of uncorrelated and seasonal systems. Physica A: Statistical Mechanics and its Applications. 2006;369(2):877–883.

4. Jiang ZQ, Xie WJ, Li MX, Zhou WX, Sornette D. Two-state Markov-chain Poisson nature of individual cellphone call statistics. arXiv preprint arXiv:150807503. 2015;.

5. Barabasi AL. The origin of bursts and heavy tails in human dynamics. Nature. 2005;435(7039):207–211.

6. Karsai M, Kaski K, Barabási AL, Kertész J. Universal features of correlated bursty behaviour. Scientific Reports. 2012;2(397). doi:10.1038/srep00397.

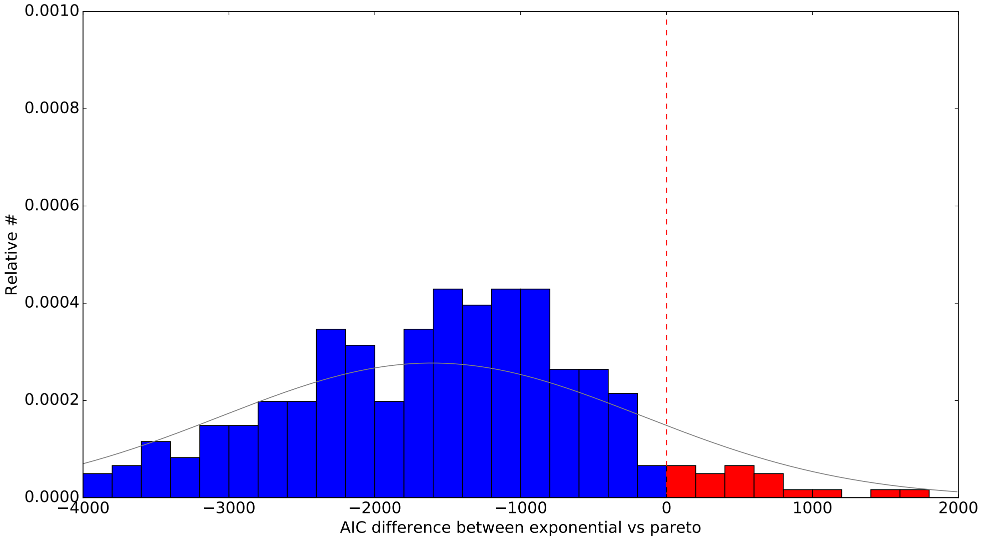

Fig 1. Histogram of the pairwise difference of the Akaike Information Criterion of a Maximum Likelihood Estimated exponential distribution vs a Pareto distribution for the inter-event times, calculated over each user of Dataset B. Blue denotes a better fit of the exponential distribution and red a better fit of the Pareto distribution. In total, for 92.6% of the users, the exponential distribution fits the inter-event times better.
