# Supplementary material for: SensibleSleep: A Bayesian Model for Learning Sleep Patterns from Smartphone Events
Source: PLoS One. 2017 Jan 11;12(1):e0169901. doi: 10.1371/journal.pone.0169901 (PMC5226832; doi:10.1371/journal.pone.0169901)
Supplement: S2 Appendix — (PDF) [file pone.0169901.s003.pdf]

S2 Goodness of Fit Asserting the goodness of fit can be done by assuming a null-hypothesis of random t_{sleep} and t_{wake} times, for each user and day, drawn from a Gaussian distribution with means and standard deviations derived from Dataset A itself, and estimate the resulting distribution of the Accuracy, Precision, Recall and F1 scores by means of a Monte Carlo simulation.

Fig 1 and Fig 2 show the distribution of this null-hypothesis (i.e. a Gaussian Simulation) vs SensibleSleep (histogram and complementary cumulative distributions).

In addition, repeating the Gaussian simulation (N=2000 times), we can estimate the distribution of the median scores achieved under the the null-hypothesis and compare it to the median score achieved by SensibleSleep. Fig. 3 shows this resulting distribution. From this, we can then estimate the probability that the Accuracy, Precision, Recall and F1 scores would under the null-hypothesis be at the achieved levels of SensibleSleep using a conventional t-test. The t-values are listed in the figure. The resulting possibility of the null-hypothesis yielding scores at the level of SensibleSleep is very low: $p < 0.00001$.

We therefore conclude that SensibleSleep provides a statistically significantly better estimate than a (weakly informative) Gaussian null-hypothesis.

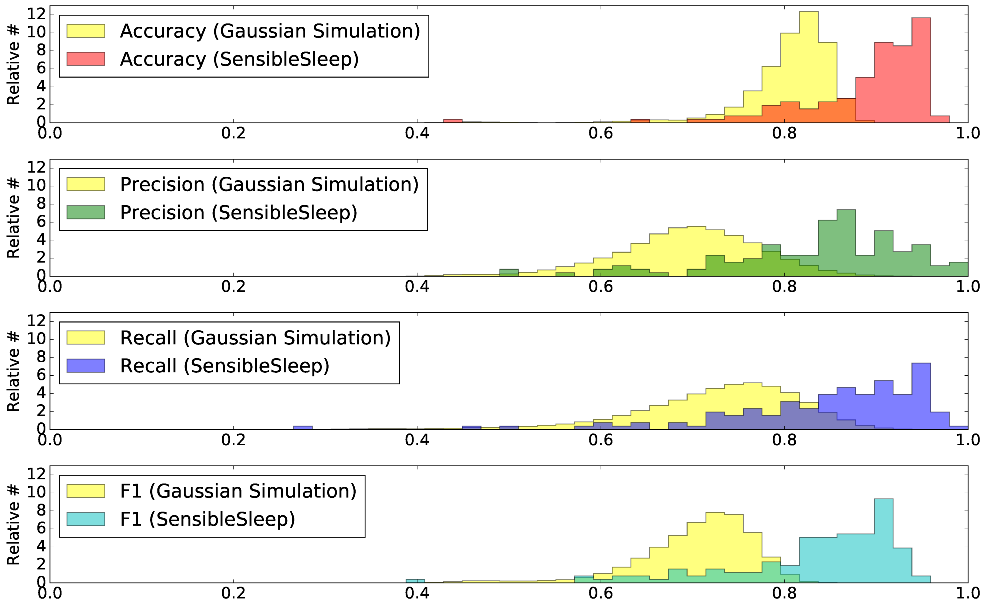

Fig 1. Accuracy, Precision, Recall and F1 scores achieved by estimating t_{sleep} and t_{wake} using a Gaussian distribution centered around the derived values (yellow) vs the scores achieved by SensibleSleep.

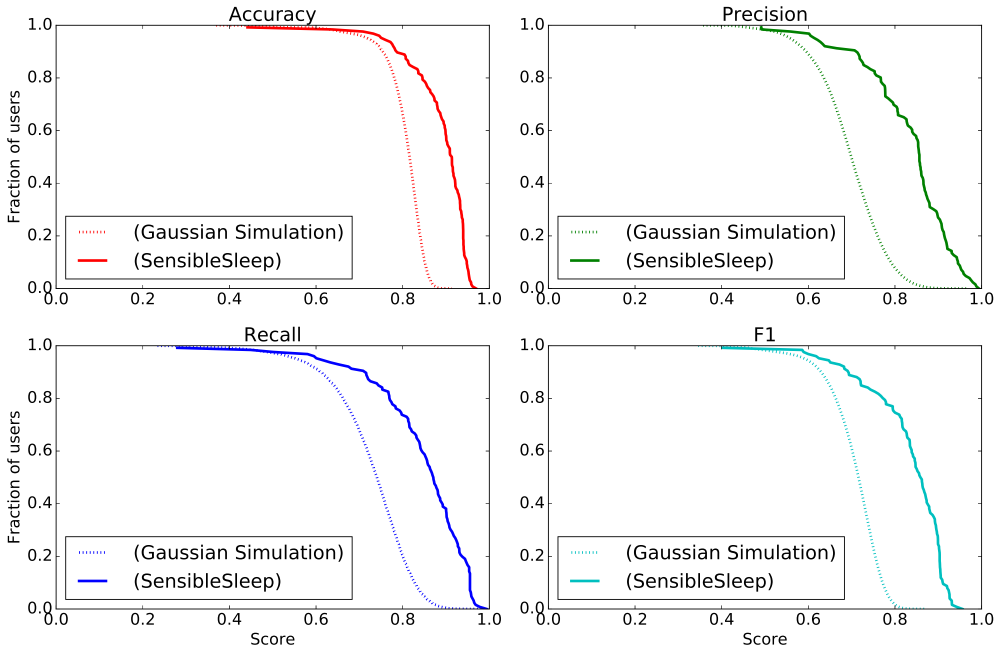

Fig 2. Complementary cumulative distriutions of Accuracy, Precision, Recall and F1 scores achieved by estimating t_{sleep} and t_{wake} using a Gaussian distribution centered around the derived values (dotted line) vs the scores achieved by SensibleSleep.

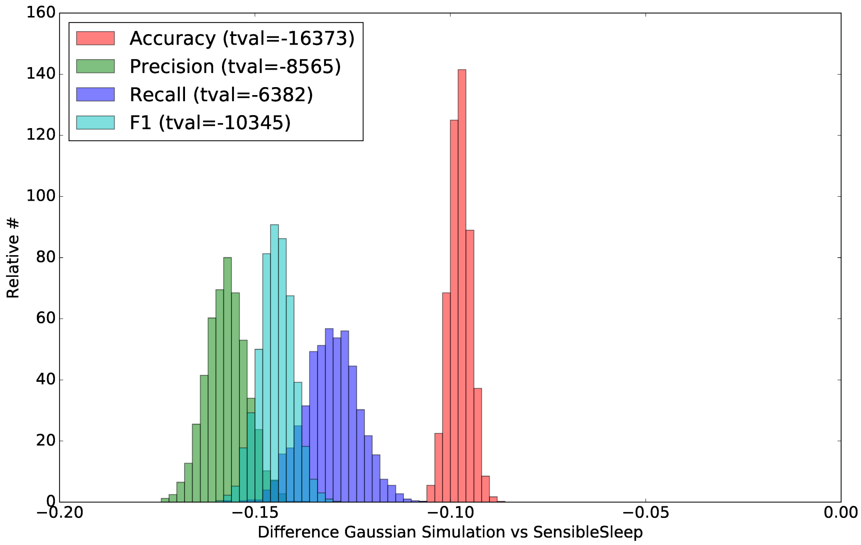

Fig 3. The difference in Accuracy, Precision, Recall and F1 scores comparing, over N=2000 runs, the median score achieved by estimating t_{sleep} and t_{wake} using a Gaussian distribution centered around the measured values vs the median score achieved by SensibleSleep. Negative values indicate worse values for the Gaussian estimator. The t-values listed show that the likelihood of achieving the scores of SensibleSleep is very low: $p < 0.000001$.
